# Characterizing Membrane Association and Periplasmic Transfer of Bacterial Lipoproteins through Molecular Dynamics Simulations

**DOI:** 10.1016/j.str.2020.01.012

**Published:** 2020-04-07

**Authors:** Shanlin Rao, George T. Bates, Callum R. Matthews, Thomas D. Newport, Owen N. Vickery, Phillip J. Stansfeld

**Affiliations:** 1 Department of Biochemistry, University of Oxford, South Parks Road, Oxford OX1 3QU, UK; 2 School of Life Sciences & Department of Chemistry, University of Warwick, Gibbet Hill Campus, Coventry CV4 7AL, UK

**Keywords:** molecular simulation, lipoprotein, membrane proteins, antibiotics, biogenesis

## Abstract

*Escherichia coli* lipoprotein precursors at the inner membrane undergo three maturation stages before transport by the Lol system to the outer membrane. Here, we develop a pipeline to simulate the membrane association of bacterial lipoproteins in their four maturation states. This has enabled us to model and simulate 81 of the predicted 114 *E. coli* lipoproteins and reveal their interactions with the host lipid membrane. As part of this set we characterize the membrane contacts of LolB, the lipoprotein involved in periplasmic translocation. We also consider the means and bioenergetics for lipoprotein localization. Our calculations uncover a preference for LolB over LolA and therefore indicate how a lipoprotein may be favorably transferred from the inner to outer membrane. Finally, we reveal that LolC has a role in membrane destabilization, thereby promoting lipoprotein transfer to LolA.

## Highlights

• Simulation of membrane association for 81 of the 114 *E. coli* lipoproteins
• The lipoprotein chaperone, LolB, has a major binding orientation with a membrane
• LolCDE deforms the membrane to initiate lipoprotein transfer
• Transport across the periplasm, from LolA to LolB, is thermodynamically favorable

## Graphical Abstract

**Figure:**
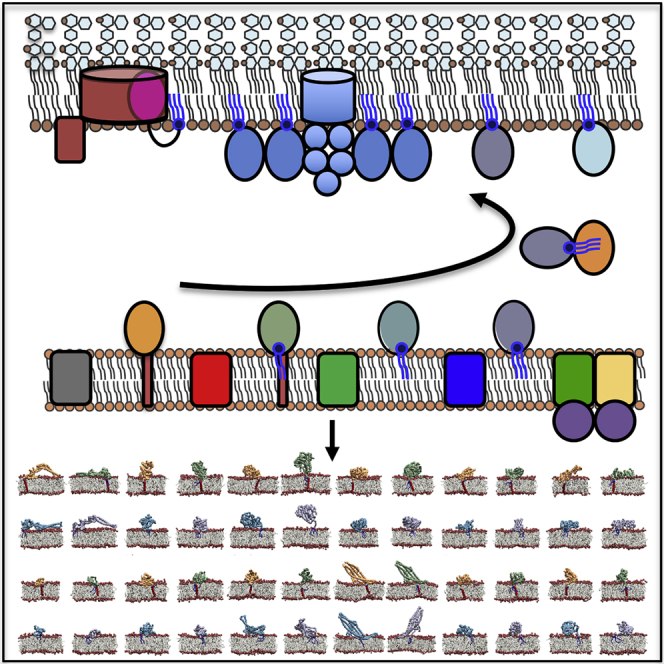

## Teaser

Rao et al. use molecular dynamics simulations to explore the lipids interactions of bacterial lipoproteins with both inner and outer membranes. They describe a workflow for performing simulations of lipid-anchored proteins, calculate the free energies for transfer of a lipoprotein from inner to outer membrane, via LolA and LolB, and hypothesize how the LolCDE mechanotransducer facilitates membrane extraction of a transported lipoprotein.

## Introduction

Anchored to the membranes of bacterial cells are a functionally diverse group of lipid-modified proteins known as bacterial lipoproteins (Madan Babu and Sankaran, 2002). They contribute, for example, to envelope stability, cell division, protein folding, signal transduction, transport, nutrient acquisition, sporulation, and conjugation, and are therefore integral to cell viability (Zuckert, 2014). Furthermore, many lipoproteins form virulence factors that actively promote surface adhesion, colonization, invasion, or immune evasion and modulation (Kovacs-Simon et al., 2011). The characteristic type of covalent lipid modifications of bacterial lipoproteins are unique to bacteria and are widely distributed across different phyla (Sutcliffe et al., 2012). The common pathway through which lipoproteins acquire their lipid anchor and reach a functional state therefore present a promising target for antibiotic development (Kitamura et al., 2018, Narita and Tokuda, 2017).

**Figure 1:**
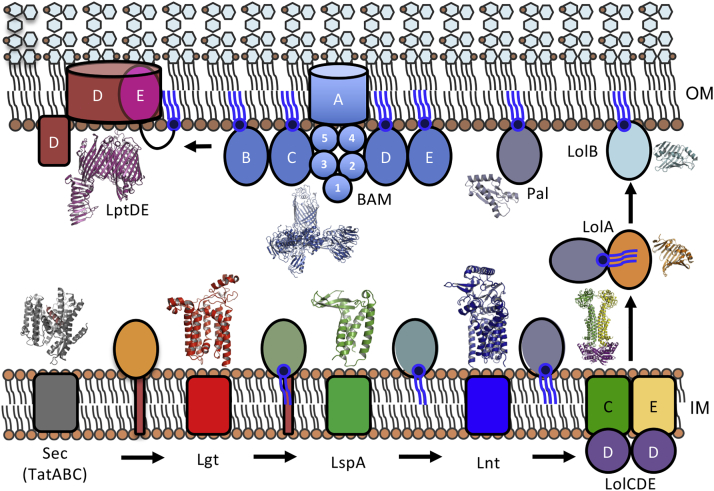
The Lipoprotein Biogenesis Pathway Sec-secreted *E. coli* lipoprotein precursors (preprolipoproteins) are sequentially post-translationally modified in the IM, by preprolipoprotein diacylglyceryl transferase (Lgt), prolipoprotein signal peptidase (LspA), and apolipoprotein N-acyltransferase (Lnt). Upon maturation, lipoproteins are either retained in the IM or transported by the Lol machinery to the inner leaflet of the OM. The Lol system is comprised of an ATP-binding cassette transporter (LolCDE), a periplasmic carrier protein LolA, and an OM receptor protein LolB that is itself a lipoprotein. Example lipoproteins Pal, BamBCDE, and LptE are shown.

Mature lipoproteins are post-translationally lipid-modified at an invariant N-terminal cysteine residue (Nakayama et al., 2012). The conventional triacylated form, consisting of two ester-linked chains and an additional amide-linked acyl group, represents a universal component of Gram-negative species. In the model system *Escherichia coli*, most known lipoproteins are associated with the inner leaflet of the outer membrane (OM) (Nakayama et al., 2012). Lipoprotein precursors (preprolipoproteins) are synthesized in the cytoplasm, with a signal peptide (SP) helix at their N terminus. They are inserted into the inner membrane (IM) by the Sec translocon and undergo three successive enzyme-mediated maturation steps. Using membrane phosphatidylglycerol as the acyl donor (Chattopadhyay and Wu, 1977), preprolipoprotein diacylglyceryl transferase (Lgt) attaches an *sn*-1,2-diacylglyceryl moiety to the conserved cysteine, located at the +1 position relative to the SP cleavage site (Sankaran and Wu, 1994). Prolipoprotein signal peptidase (LspA) then cleaves the transmembrane (TM) SP, leaving an apolipoprotein anchored to the membrane only via its two acyl tails (Dev and Ray, 1984). Finally, apolipoprotein N-acyltransferase (Lnt) catalyses the addition of a third chain, converting the diacylglycerylcysteine to triacylglycerylcysteine (Gupta and Wu, 1991) (Figure 1).

Upon maturation, triacylated lipoproteins that are destined for the OM, i.e., those lacking a retention signal, are delivered across the periplasm through the lipoprotein OM localization (Lol) pathway (Tokuda and Matsuyama, 2004). The LolCDE complex, an ATP-binding cassette (ABC) transporter, extracts the lipoprotein substrate from the IM (Yakushi et al., 2000). Powered by energy from ATP hydrolysis in the LolD ATPase homodimer, the lipoprotein molecule is transferred from the LolE subunit to a soluble carrier protein, LolA (Matsuyama et al., 1995), which is recruited by LolC (Kaplan et al., 2018). The disengaged lipoprotein:LolA complex traverses the periplasm to reach the OM where the receptor protein LolB—itself a lipoprotein—accepts a LolA-bound lipoprotein and incorporates it into the inner leaflet (Konovalova and Silhavy, 2015, Matsuyama et al., 1997). The genes encoding the components of this maturation and localization pathway, including all three enzymes and the Lol proteins, are essential for *E. coli* growth (Buddelmeijer, 2015).

The structural basis for the machinery associated with lipoprotein processing and transport was, until recently, limited. Molecular details of Lgt and LspA were first described at the start of 2016 (Mao et al., 2016, Vogeley et al., 2016), with structures of Lnt and periplasmic domains of LolC and LolE solved the following year (Crow et al., 2017, Wiktor et al., 2017). A complete structure of the LolCDE complex has yet to be determined experimentally; however, its sequence homology with the recently determined MacB structures yield the comparative coordinates for the IM transporter (Crow et al., 2017, Fitzpatrick et al., 2017, Okada et al., 2017). Structures of the soluble chaperone proteins, LolA and LolB, have been known for over a decade; however, the coupling between LolA and the periplasmic domain of LolC was only described in full atomic detail in 2018 (Kaplan et al., 2018).

Crystal structures of LolA and the protein portion of LolB reveal remarkable structural similarity despite their low sequence homology (Takeda et al., 2003). Both proteins have a hydrophobic cavity composed of an unclosed β-barrel and an α-helical lid, creating a potential binding site for the lipid moiety of lipoproteins. Structural and spectroscopic studies on LolA, employing an R43L mutant where critical interactions that stabilize cavity closure are eliminated, have shown opening and closing of the LolA lid upon lipoprotein binding and release (Oguchi et al., 2008). Solved LolB structures, however, are in a conformation where the barrel entrance is obstructed, and the calculated cavity size is insufficient to accommodate multiple acyl chains (Takeda et al., 2003). Nevertheless, lipid-binding roles of the hydrophobic cavities are also supported by NMR and photo-crosslinking analyses of LolA:LolB interactions (Nakada et al., 2009, Okuda and Tokuda, 2009). Unidirectional lipoprotein transfer from LolA to LolB has been indicated to be driven solely by an increase in affinity, with the lipid moiety interacting more favorably with LolB than LolA (Taniguchi et al., 2005). However, the mode and energetics of interaction between a lipoprotein lipid moiety and either LolA or LolB remain to be characterized.

In addition to the structural details of the lipoprotein biogenesis machinery, there have also been recent advances in the determination of the structures of bacterial lipoproteins themselves. Identification of bacterial lipoproteins is predominately enabled by the presence of the conserved lipobox motif ([LVI][ASTVI][GAS][C]) in the amino acid sequence (von Heijne, 1989). Using this motif, over 2,000 entries have been annotated, under the PROSITE pattern PS51257, in the UniProtKB/Swiss-Prot database (http://www.uniprot.org/) (Bairoch et al., 2005, Sigrist et al., 2002). At the time of writing, 165 of the 2,196 UniProtKB entries have, at least partially, been structurally characterized by either X-ray diffraction, NMR spectroscopy, or cryo-electron microscopy (cryo-EM).

Lipoproteins are frequently components of larger macromolecular complexes, for which there are now structural details. These include the OM protein translocon, BamABCDE (Bakelar et al., 2016, Gu et al., 2016), the lipopolysaccharide (LPS) transporter, LptDE (Botos et al., 2016, Dong et al., 2014), and the biological construct used for DNA sequencing by nanopore technologies, CsgG (Goyal et al., 2014). Despite these advances, most of the structures lack full details for the triacylated cysteine and the N-terminal linker. Examples where the lipidated cysteine has been resolved include the heavy metal efflux pump, CusC, the capsular polysaccharide exporter (Kulathila et al., 2011), Wza (Dong et al., 2006), and a subunit of the Alternative Complex III, ActC (Sun et al., 2018). Although X-ray diffraction and single-particle cryo-EM methods have captured the lipid tail, NMR has in a few instances been used to capture the structural dynamics of the N-terminal linker, for example, LpoB (Egan et al., 2014), YajI, and YehR. Nevertheless, these examples remain a small fraction, with most lipoprotein structures only capturing the folded core of the protein.

In this study, we have developed a pipeline to model the N-terminal section onto the lipoprotein structures, incorporating one of the four stages of maturation within the model. To achieve this, we have prepared both coarse-grain (CG) and atomistic parameters for the diacyl- and triacylcysteine post-translational modifications, and formulated a means to switch between the two. We have combined this with our computational pipeline, MemProtMD (http://memprotmd.bioch.ox.ac.uk/) (Newport et al., 2019, Stansfeld et al., 2015), to enable the *in silico* reconstitution of the modeled lipoproteins within a lipid bilayer environment. This enables the evaluation of the functional dynamics of lipoproteins in their partially or fully lipidated states in association with the membrane.

**Figure 2:**
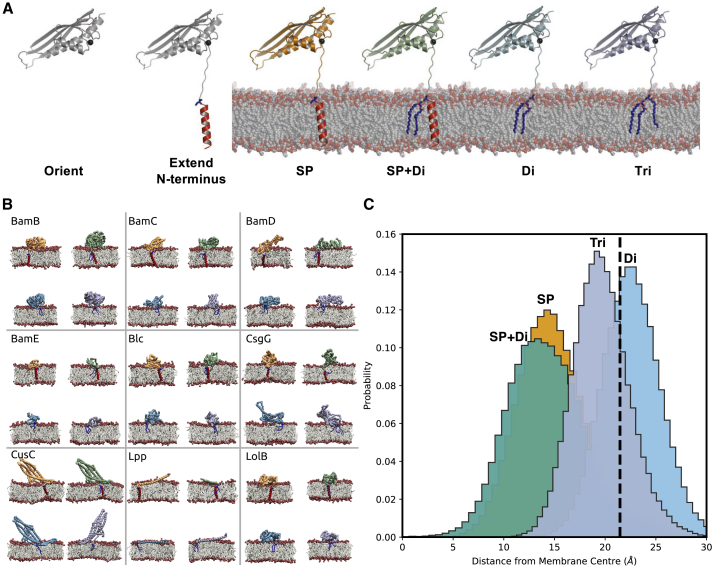
Workflow for Modeling and Simulating the Four Stages of Bacterial Lipoprotein Biogenesis (A) The structure of a lipoprotein is reoriented so that the N-terminal end is positioned closest to the membrane. A flexible tether is modeled onto the structure, with SP, SP and diacylcysteine (SP + Di), diacylcysteine (Di), or triacylcysteine (Tri) modification. The states were subjected to CG-MD simulations. (B) CG-MD snapshots for a subset of the 30 molecular structures of *E. coli* lipoproteins. The membrane association with the four discrete membrane anchors is shown for each lipoprotein. In each case, the membrane association of the lipoprotein is shown at the end of a 1 μs simulation, with either its SP (top left), SP + Di (top right), Di (bottom left), or Tri modification (bottom right). (C) Membrane localization of the conserved cysteine residue in the four stages of maturation.

We demonstrate our approach with a subset of 30 *E. coli* lipoproteins for which PDB structures are available (Berman et al., 2007) (Figure 2) and then extend this methodology with the aim of capturing structures and membrane associations for all 114 predicted *E. coli* K12 lipoproteins.

We then focus on the cysteine-lipid moiety, using the developed lipoprotein parameters to quantify the strength of its binding to both membranes and to the transfer proteins, LolA and LolB. From this we derive an energetic basis for the lipoprotein localization pathway, by calculating potential of mean force (PMF) free energy profiles through series of umbrella sampling molecular dynamics (MD) simulations, a technique that has been previously employed for the characterization of lipid-protein and lipid-membrane interactions (Arnarez et al., 2013a, Arnarez et al., 2013b, Hedger et al., 2016).

Finally, we assess the membrane dynamics around a model of the LolCDE complex with the aim of conceptualizing its role in lipoprotein extraction from the bacterial IM.

## Results

There are 114 predicted lipoprotein sequences in the widely used lab strain of *E. coli* K12, of which 30 proteins have been structurally elucidated by X-ray diffraction, NMR, or cryo-EM to yield appropriate molecular structures for our initial round of modeling (Table S1). All lipoproteins are initially secreted into the periplasm with a single-pass TM helix anchoring it to the membrane. This initial stage of maturation was used as the scaffold in our methodology for the three other states (Figures 2 and S1).

### Lipoprotein Maturation States Stably Reconstituted into Membranes

For each of the 30 *E. coli* lipoproteins, four CG (Marrink et al., 2004, Monticelli et al., 2008) molecular models were created at successive stages along their modification pathway: unlipidated preprolipoproteins, diacylated prolipoproteins, apolipoproteins, and triacylated mature lipoproteins (Figures 2A and S2). For comparison, we also simulated each system without either protein or lipid tether. The 5 × 30 models were then individually reconstituted into a preformed bilayer environment that consisted of the membrane phospholipids POPE and POPG (i.e., 1-palmitoyl-2-oleoyl-*sn*-glycero-3-phosphoethanolamine and -phosphoglycerol) in a 4:1 ratio, approximating the major components of *E. coli* membrane: 1 μs of simulation data was then obtained for each lipoprotein-membrane system (Figure S3).

Of the experimentally derived structures, LolB reveals a consistent binding mode with the membrane in its four maturation states (Figures 2B, S3, and S4). This likely reflects the nature of its own involvement in the transfer of a lipoprotein from LolA into the OM. In addition to LolB, the lipoproteins YceB, Blc, CusC, Lpp, and LpoA all form extremely consistent membrane interactions irrespective of the maturation state (Figures 2B, S3, and S4). For LolB, Blc, and CusC, these interactions appear to be independent of the molecular tether, with strong membrane interactions maintained without either lipid or protein anchor. In contrast, the removal of the tether attached to YceB, Lpp, and LpoA greatly reduces the correlation of the protein-lipid contacts with any other state. At the other end of the spectrum, CsgG does not form comparable contacts in any maturation state, suggesting its membrane interactions are inconsequential before its nonameric pore-forming state (Figure 2B). The interactions of RcsF with the membrane are also varied, which may be reflective of its lengthy tether and also its likely role in protein-protein interactions rather than direct membrane contact.

### Assessment of the Mature Triacylated Lipoproteins

**Figure 3:**
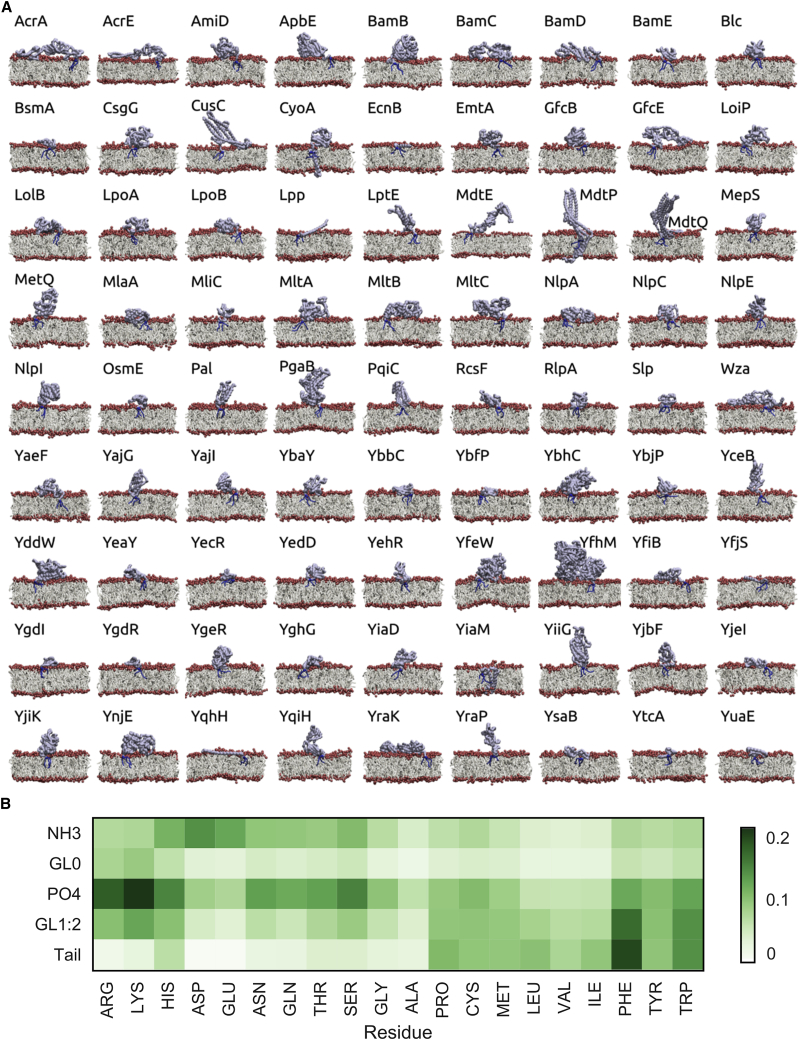
Membrane Associations of Lipoproteins (A) Exemplar membrane interactions of the 81 triacyl-modified lipoproteins bound to a model IM, before their translocation across the periplasm to the OM. (B) Residue-lipid contacts for bacterial lipoproteins. Assessment of the residue interactions with the glycerol group of POPG (GL0), ethanolamine group of POPE (NH3), phosphate groups (PO4), glycerol (GL1:2), and acyl tails (Tail) of both lipids.

**Figure 4:**
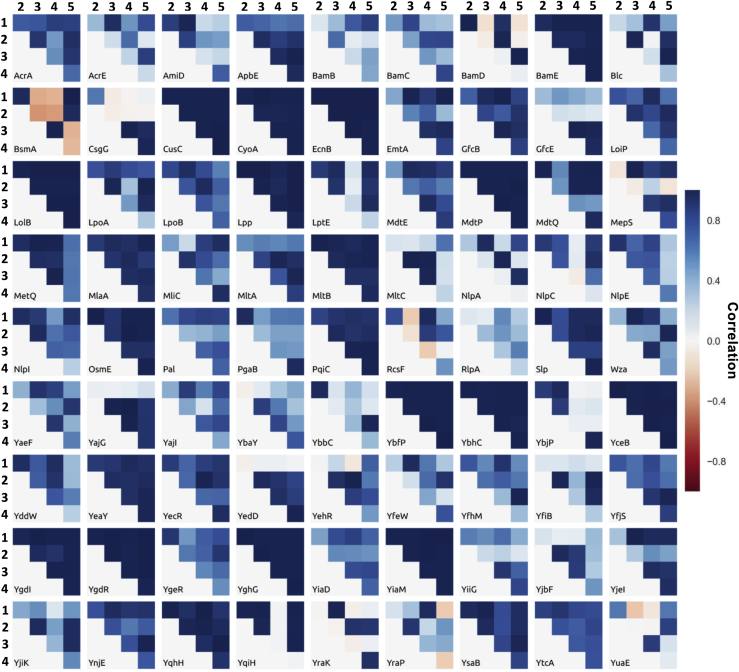
Reproducibility of Membrane Association of the 81 Modeled Triacylated *E. coli* Lipoproteins For each of the 81 modeled lipoprotein structures the similarity in the membrane-protein interactions are compared in the five repeats of membrane association. A dark blue color reflects faithful reproduction in binding mode between two simulations and a Pearson correlation coefficient of 1, while a red color highlights a distinct binding orientation and a Pearson correlation coefficient of -1 for the lipid-residue interactions. The five simulations per lipoprotein are labeled 1 through 5.

Although all lipoproteins are lipid-tethered to retain their membrane proximity within the periplasm, it was of interest to discover whether lipoproteins form well-defined membrane interactions. Here, we show that ∼25% of the 81 triacylated systems simulated formed a reproducible interface with the membrane (Figures 3A and 4). As with the maturation states, CusC, LolB, Lpp, and YceB formed reproducible membrane interactions. Other consistent binders include YghG, BamE, EcnB, MdtP, MlaA, MltB, OsmE, PqiC, Slp, YbfP, YbhC, YeaY, YdgI, YdgR, YghG, YqhH, YsaB, and YtcA, and the two TM helix-containing lipoproteins, YiaM and CyoA. For this set of simulations, the membrane interactions of Blc and LpoA are only partially reproducible, with additional membrane-binding modes observed (Figure 4).

**Figure 5:**
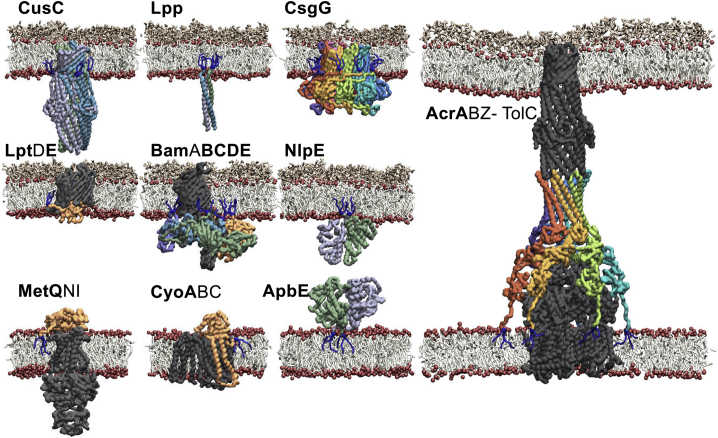
Molecular Simulations of Lipoprotein Complexes The methodology allows for the construction of lipoprotein tethers within macromolecular complexes; here shown for ten structures of *E. coli* K12 lipoprotein complexes, CusC, Lpp, CsgG, LptDE, BamABCDE, and NlpE in a model OM, MetQNI, CyoABC, and ApbE in a model IM, and the AcrABZ-TolC complex spanning the periplasm and inserted into both IM and OM. In each case the triacyl lipoprotein tether is shown in blue sticks, with the lipoprotein colored. The non-lipoprotein subunits are shown in gray.

From the ensemble of simulations, we are able to identify the primary amino acids that contact the lipid membrane, upon lipoprotein association (Figure 3B). From this analysis, phenylalanine stands out as a major interaction partner with both the lipid tails and glycerol linkage, anchoring into the membrane core. As previously observed for integral membrane proteins (Newport et al., 2019), arginine and lysine interact with the phosphate group and have a degree of selectivity for the glycerol moiety of the phosphatidylglycerol-containing lipids. Conversely, aspartate and glutamate are the major interaction partners for the ethanolamine head groups (Figure 3B). As a number of lipoproteins form complexes in their mature state, we also modeled and simulated these macromolecular systems to test the flexibility of the protocol (Figure 5).

### A Focus on Periplasmic Transport

**Figure 6:**
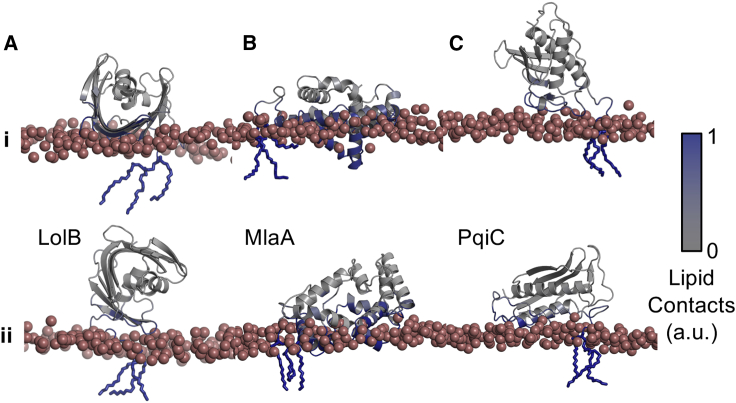
Lipoproteins Involved in Periplasmic Transport Membrane association of (A) LolB, (B) MlaA, and (C) PqiC in their (i) primary and (ii) secondary binding orientations. Phosphate atoms are shown as red spheres. Proteins are shown as a cartoon representation, and colored on a white to blue scale, with blue indicating extensive lipid contacts. See also Supplemental Information, Figure S5.

The reproducible binding for LolB is suggestive of the importance of its orientation for lipoprotein localization. In addition, two other lipoproteins within our dataset have a role in the periplasmic transfer of lipid-like molecules, MlaA and PqiC. These proteins also reveal a reproducible binding mode with the membrane, in the five replicates. We therefore expanded the simulation dataset for all three molecular systems to 25 repeats. All three cases reveal further instances of the primary binding mode to the membrane, with secondary binding modes also elucidated in the larger datasets (Figure 6).

The primary binding mode of LolB rests the convex surface of its β-barrel upon the lipid head groups, while its secondary binding mode is rotated roughly 90° with respect to the membrane (Figures 6A and S5A). Both binding modes are anticipated to be well-suited to enable the transfer of the triacyl lipoprotein tails into the membrane. The primary binding mode of MlaA penetrates deep into the membrane, comparable with the bilayer depth previously observed in its complex with OmpF (Abellon-Ruiz et al., 2017, Yeow et al., 2018) (Figures 6B and S5B). This mode of monotopic membrane insertion induces deformation of the membrane and in all of the simulations we observe lipid capture within its central pore. For this lipoprotein, the secondary binding mode is largely equivalent to the first in all but the depth of its membrane penetration, resting further into the solvent phase and, therefore, may reflect a transitional binding mode, before monotopic membrane insertion. The analogous lipid transport lipoprotein, PqiC, also forms well-defined and reproducible contacts with the membrane (Figures 6C and S5C). Unlike MlaA it does not penetrate into the membrane, rather resting on its surface. This is an appropriate orientation for multimerization of the octameric PqiC ring. In a second binding pose, observed in only three of the simulations, PqiC lies flat on the surface of the membrane.

We use these three examples to illustrate the multiscale aspect of the pipeline, by converting both major and minor binding orientations of the three lipoproteins to a CHARMM36 resolution and performing three repeats each of 100 ns atomistic simulation (Figure S5). In all three instances the primary binding modes are retained, with MlaA showing a greater degree of membrane deformation and lipid transport at the atomistic resolution (Figure S5E). In the secondary binding modes, LolB tumbles to make further membrane contacts, while both MlaA and PqiC retain the binding orientations from the start of the 100 ns simulation.

### Interactions of the Triacyl Moiety with LolA and LolB

**Figure 7:**
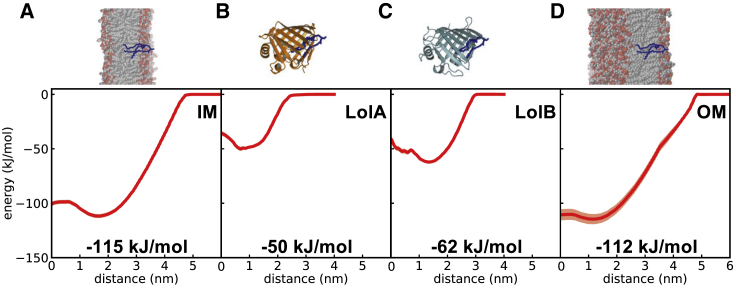
Calculating the Energetics of Lipoprotein Transport The energetics associated with lipoprotein transfer across the periplasm, obtained from umbrella sampling and PMF calculations, calculated using WHAM with errors computed using Bayesian bootstrapping. Plots are shown for the extraction of the triacylated cysteine moiety from (A) IM, (B) LolA (orange), (C) LolB (cyan), and (D) OM. The depicted energy values are derived from the minimum value of the PMF.

The open conformation of LolA R43L mutant (PDB: 2ZPD) (Oguchi et al., 2008) was used as the scaffold for the open states of both the wild-type LolA and LolB structures. In the case of the latter, the closed structure of LolB (PDB: 1IWM) was structurally aligned and used to guide the conformational rearrangement (Takeda et al., 2003). Preliminary CG simulations were configured and performed with solvated LolA and a single unbound triacylcysteine. Over the course of a 2 μs simulation the triacylated cysteine approached and inserted itself into the hydrophobic cavity of LolA, with a relatively stable configuration of the triacylcysteine established and maintained through the duration of the simulation. In this configuration, the amino acid backbone of the cysteine is situated toward the mouth of the cavity, with the three lipid tails buried into the core of the LolA cavity (Figure 7). This arrangement is suitable for connection of the lipid moiety to the remainder of a mature lipoprotein. In this configuration, the bound triacylcysteine overlaps the “hook” loop of LolC that was observed in the LolA:LolC complex (Kaplan et al., 2018), suggesting that the hook will likely be displaced before triacyl binding as part of the lipoprotein localization mechanism.

### Free Energy of Cysteine-Lipid Moiety Interactions with LolA, LolB, and the Bilayer

For the purpose of the free energy calculations the bound configuration of the triacyl from the MD simulations was idealized in the LolA and LolB cavities based on the coordinates from the preliminary MD simulations. Umbrella sampling MD simulations (Domanski et al., 2017, Hedger et al., 2016) were performed to calculate PMF free energy profiles. To compute the PMF for both LolA and LolB, triangle position restraints were applied to the base of the proteins. To test that these restraints had limited impact on the overall protein dynamics we performed three repeats of 1 μs simulations for the triacylcysteine bound and apo states, with and without the restraints. Limited differences were observed in the root-mean-square fluctuations of the simulations with and without restraints and, therefore, they should have limited impact on the computed PMF (Figure S6).

The extraction of a solitary triacylated cysteine into an aqueous environment required a sizable energy input of approximately 115 kJ/mol (Figure 7A). Relative to this high-energy state, the energy for extraction of the cysteine-lipid moiety from inside LolA or LolB were recorded at 50 and 62 kJ/mol, respectively (Figures 7B and 7C). These values are comparable with those recently reported for LolA with a bound antibiotic (Boags et al., 2019). The energy required for extraction of the triacylated cysteine from a model OM was slightly less than that from the IM, at 112 kJ/mol, which is within the error of the free energy calculated for the IM. This may also be due to the thinner hydrophobic core of the OM, induced by the shorter-tailed LPS molecules that comprise the outer leaflet (Figure 7D). All free energy values quickly converged within the three replicas of the 1 μs simulations and showed good histogram overlap (Figures S7 and S8).

We also evaluated the free energies of extraction of the diacylcysteine. In all instances the diacyl moiety had a reduced binding affinity with respect to the triacyl form; however, the same trend was received. To extract the diacyl group from a membrane required 101 kJ/mol. Similar values were received for LolA and LolB, respectively, 53 and 55 kJ/mol, while the value for OM removal was 95 kJ/mol (Figure S7).

### LolC Induces Membrane Deformation Enabling Lipoprotein Export

**Figure 8:**
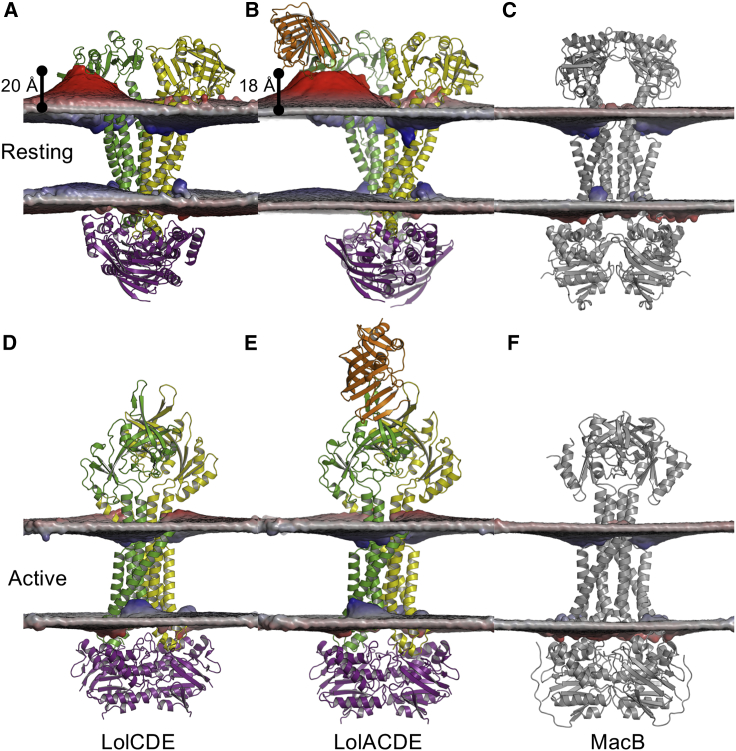
Molecular Simulations of the Lol and Mac Transporter Complexes In the resting state, LolCDE (A) and LolACDE (B) complexes reveal extensive membrane deformation about the periplasmic domain of LolC, with the position of the phosphates changing by up to 20 Å from their bulk membrane position. The equivalent state of MacB (C) does not show membrane deformation, nor do simulations in the active, ATP-bound states of (D) LolCDE, (E) LolACDE, or (F) MacB. Proteins are shown in a cartoon representation, highlighting LolC (green), LolD (purple), LolE (yellow), LolA (orange), and MacB (gray). Cumulative phosphate positions from the simulations are shown as a surface, on a red-white-blue scale, from thickening to thinning of the membrane.

Molecular models of the LolCDE transporter were built based on the MacB structures in their resting (PDB: 5WS4) and activated (PDB: 5LIL) states. In the resting state the proximity of the periplasmic domain of LolC to the outer leaflet induces significant membrane deformation, with up to 42 Å between the membrane center and the phosphtate groups; this is relative this is to a bulk membrane thickness of 22 Å (Figure 8). This level of deformation is not observed in the activated, ATP-bound state, nor do we observe distortion about the LolE periplasmic domain. This therefore appears to be stimulated by the proximity of the LolC periplasmic domain to the membrane, with contacts between membrane and the soluble domain stabilizing a tilt of the ABC transporter (Figure S9).

We compare this level of deformation with a model of the LolCDE complexed with LolA, based on the crystal structure of LolC bound to LolA (Kaplan et al., 2018). As before, a reproducible membrane deformation is observed about the LolC periplasmic domain, extending to 39 Å from the membrane center, in close proximity to the “mouth” of LolA. No membrane deformation is observed in the activated form of these complexes. We relate this with simulations of the MacB transporter structures in both states. In neither case is membrane deformation observed, suggesting that the deformation is symptomatic of the LolC subunit when in close proximity to the membrane, and is illustrative of a mechanism for facilitating lipoprotein extraction from the IM. Therefore, this degree of membrane deformation appears to be unique to the LolCDE transporter. To the best of our knowledge very few other membrane protein structures induce such local membrane curvature in the ∼4,000 membrane protein simulations currently housed in MemProtMD (Newport et al., 2019).

We tested this further by running atomistic simulations, based on the coordinates from the CG simulations, for LolCDE and LolACDE complexes in both resting and activated states. Regarding the CG simulations, the membrane deformation is only observed for the resting state complexes, with the membranes remaining planar in the activated forms of the transporter (Figure S9).

## Discussion

Here, we present a pipeline for modeling and simulating a crucial and ubiquitous set of bacterial peripheral membrane proteins that undergo four levels of maturation. These stages directly impact on the mode by which the lipoproteins are anchored to the membrane, and it is therefore of interest how this influences the interactions made with the membrane. Here, we have simulated the mode of membrane binding at each maturation stage for 30 lipoprotein structures, before then extending the methodology to incorporate 81 models of the 114 predicted *E. coli* K12 lipoproteins in their triacylated, mature state. The results illustrate the relative consistency of membrane binding for each entry and provide a structural and simulation dataset for lipoproteins expressed by *E. coli* K12 cells. Ultimately, we will aim to extend this dataset to capture the 33 lipoprotein structures not represented here, while also including key lipoproteins from other species of bacteria.

We demonstrate the importance of this methodology through molecular simulations of triacyl versions of LolB, MlaA, and PqiC. In each case, the lipoproteins form well-defined membrane interactions that are likely important in their mechanism of transport of lipid moieties. Of particular interest to this overall study are the two identified binding orientations of LolB, with the convex face of the β-barrel forming a tight interface with the bilayer to maintain LolB in close proximity to the membrane, in two distinct configurations.

**Figure 9:**
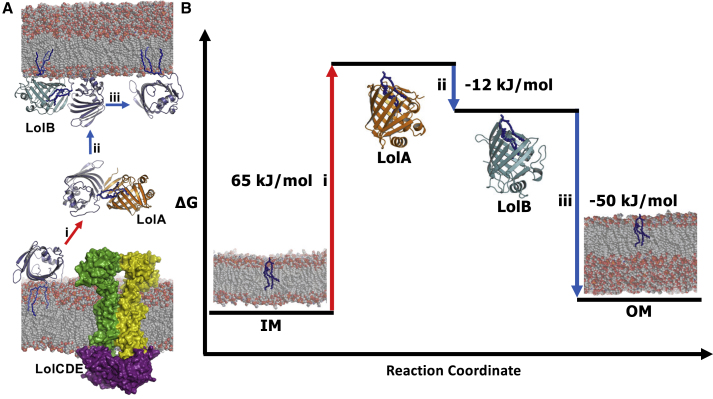
Thermodynamics of Lipoprotein Transfer (A) Structural basis for transport of triacylated LolB (blue)—itself a lipoprotein—from the IM to OM via LolA (orange) and LolB (cyan). (B) Thermodynamic cycle of lipoprotein transport, combining the values from the PMF calculations in Figure 7. The energy needed for membrane extraction is obtained from ATP binding and hydrolysis by LolCDE. Once extracted from the IM the bioenergetics of transport is downhill from LolA to LolB to the OM.

The present study also offers an estimate of the energetic changes underlying the three steps of lipoprotein localization, under the assumption that hydrophobic interactions with the lipid anchor constitute a predominant amount of binding free energy at each stage. The cysteine-lipid moiety of mature lipoproteins confers a high level of membrane-binding strength, approximately equivalent to the insertion of a TM helix. We estimate, through pairwise subtraction, that the energy requirement of the first step of the pathway, whereby the triacyl lipid is extracted from the IM and forms a complex with LolA, is +65 kJ/mol (Figure 9). As the ΔG_hydrolysis_ values for ATP range from approximately −31.55 kJ/mol (Meurer et al., 2017) to −46.5 kJ/mol (Tran and Unden, 1998) per ATP molecule, this value for free energy of triacyl transfer approximates to the expected free energy released upon hydrolysis of two ATP molecules by the LolCDE transporter. We anticipate that the significant impact the LolCDE transporter imparts on the stability of the IM will also reduce the energy requirement for triacyl extraction. Furthermore, as this deformation is driven by the periplasmic domain of LolC it is indicative of a pathway for direct lipoprotein triacyl transfer from the outer leaflet of the membrane to its binding site within LolA.

The mechanism for this type VII mechanotransduction system, with its LolA chaperone, is distinct from the type VI class of ABC transporters, for which we have recent structures of LptBFG bound to the transported LPS (Li et al., 2019, Owens et al., 2019). In the case of LptBFG, the LPS molecule binds central to the transporter core, with the carrier proteins expected to form a continuous chute to LptDE in the OM (Dong et al., 2014).

Our simulation results further corroborate the lipid-binding capacities of LolA and LolB. Contrary to previous descriptions (Narita and Tokuda, 2017), the hydrophobic cavities of both proteins are indicated to be capable of accommodating multiple acyl chains, with quantitative confirmation that the transfer from LolA to LolB (ΔG_transfer_ approximately −12 kJ/mol) is driven by an increase in affinity (Taniguchi et al., 2005) (Figure 9). Once bound to LolB, the triacyl is then delivered to the membrane, through the tight association that LolB has with the inner leaflet of the OM, and coupled to a favorable ΔG_transfer_ of approximately −50 kJ/mol (Figure 9). A comparable thermodynamic funnel has been observed for LPS transfer in the TLR4 pathway (Huber et al., 2018). Ultimately this means that the thermodynamic cycle ends at a higher state than the start, with an energetic cost of ∼3 kJ/mol per lipoprotein transferred, although this is within the error of the calculations (Figure 9).

The diacylcysteine moiety also follows a similar trend for periplasmic transport, yet only triacyl lipoproteins are expected to be transported. Therefore, selectivity must occur at the stage of interactions with the LolCDE transporter, with the unmodified N-terminus of the diacylcysteine expected to carry a +1 charge under standard conditions.

### Conclusions

By developing a simulation pipeline for membrane-anchored lipoproteins, we have evaluated each stage of their maturation, hypothesized a mechanism for their membrane extraction, and elucidated the thermodynamic basis of their transport across the periplasm to the bacterial OM. This study yields a candidate mechanism for direct transfer of a triacyl lipoprotein from the IM to LolA through membrane budding. We envisage that our results will promote further studies into how mechanistically the Lol system operates, for example how it discriminates between tri- and diacyl-lipoproteins; including those that contain IM retention signals.

## STAR★Methods

### Key Resource Table

**Table:** 

REAGENT or RESOURCE	SOURCE	IDENTIFIER
**Software and Algorithms**		

Gromacs 2018 and 2019	(Abraham et al., 2015)	www.gromacs.org
Martini force field v2.2 and v3	(de Jong et al., 2013)	www.cgmartini.nl
CHARMM36 force field	(Klauda et al., 2010) (Huang et al., 2017)	mackerell.umaryland.edu/charmm_ff.shtml#gromacs
CHARMM-GUI	(Jo et al., 2017)	www.charmm-gui.org
Pymol 1.8	(The PyMOL Molecular Graphics System, 2015)	pymol.org
VMD 1.9.2	(Humphrey et al., 1996)	www.ks.uiuc.edu/Research/vmd
Modeller 9.16	(Sali and Blundell, 1993)	salilab.org/modeller/
SWISS-MODEL	(Waterhouse et al., 2018)	swissmodel.expasy.org
Gremlin Database	(Ovchinnikov et al., 2014)	gremlin.bakerlab.org/complexes.php
Phyre2	(Kelley and Sternberg, 2009)	www.sbg.bio.ic.ac.uk/∼phyre2/
INSANE	(Wassenaar et al., 2015)	www.cgmartini.nl/index.php/downloads/tools/239-insane
MemProtMD	(Stansfeld et al., 2015)	memprotmd.bioch.ox.ac.uk
MDAnalysis	(Michaud-Agrawal et al., 2011)	www.mdanalysis.org

### Lead Contact and Materials Availability

Further information and requests for materials or resources should be directed to and will be fulfilled by the Lead Contact, Phillip Stansfeld (phillip.stansfeld@warwick.ac.uk). This study did not generate new unique reagents.

### Experimental Model and Subject Details

No experimental models were used in this study.

### Method Details

#### Identifying Lipoprotein Structures from *E. coli*

The PROSITE pattern PS51257 proposes that that there are 108 protein sequences in *E. coli* K12 that possess a lipobox motif and are therefore likely to be a bacterial lipoprotein. Four further protein sequences are predicted to be post-translationally modified, but not detected by PROSITE. Two more proteins, YfhG and YnbE, are likely to be lipoproteins, but are not captured by the previous search term, taking the number of predicted sequences to 114. Of these proteins, 38 have been structurally elucidated by either X-ray diffraction, NMR or cryo-EM (Supplemental Information, Table S1). Two further lipoprotein structures, Wza and YjiK, have been resolved from other strains of *E. coli*. Of the 38 K12 structures, eight were deemed inappropriate for our initial round of modelling: CyoA is complicated by two TM helices, MltD and YiaD lack coordinates for their N-terminal domains, while MepS, MltB, Pal, LpoB and YfhM have N-terminal linkers that are greater than 35 residues in length to their structured core domain. All but LpoA – which lacks its C-terminal domain in the X-ray structure – are simulated as the near-complete form of the protein.

#### Extending the Methodology to all 114 *E. coli* K12 Lipoproteins

The methodology was then broadened to build models for all hypothetical *E. coli* K12 lipoproteins. In addition to the 38 lipoprotein structures previously described, Phyre2 was used to detect homologous structures, with the aim of building comparative models for all 114 lipoproteins (Kelley and Sternberg, 2009). Co-evolution data was also explored, with a Rosetta-folded model of YiaM incorporated into our dataset and used in our simulations (Ovchinnikov et al., 2014). It was not possible to construct coordinates, beyond fragments, for 21 of the lipoprotein sequences, with 12 further structures missing greater than 70 N-terminal amino acids in their experimental coordinates. Therefore, 81 lipoprotein structures could be accurately modelled and were subjected to 5 repeats of MD simulation to assess their membrane association, orientation and interactions as mature lipoproteins with a triacyl tether (Figures 3 and 4 and Table S1).

#### Incorporation of Triacyl Tethers into Lipoprotein Complexes

Lipoproteins are also components in key homo- and heteromeric macromolecular complexes. To date there are ten distinct *E. coli* K12 complexes for which we have structures: BamABCDE (Gu et al., 2016), LptDE (Dong et al., 2014), MetQNI (Nguyen et al., 2018), CusC (Kulathila et al., 2011), Lpp (Liu et al., 2003), ApbE (Deka et al., 2016), NlpI (Wilson et al., 2005), CsgG (Goyal et al., 2014), CyoAB (Abramson et al., 2000), and AcrABZ-TolC (Wang et al., 2017) (Figure 5). Of these complexes, four contain lipoproteins that are retained in the IM, CyoA, MetQ, ApbE and AcrA, with AcrA part of a super-complex that spans the entirety of the cell envelope. To illustrate that this methodology is also appropriate for lipoprotein complexes, we constructed and simulated OM-expressed lipoproteins in a model OM bilayer (Hsu et al., 2017a), the ApbE dimer and CyoAB in a model *E. coli* IM and AcrABZ in an IM, connected across the periplasm by TolC in the OM (Hsu et al., 2017b).

#### Modelling of Lipid Moieties

CG models for the cysteine-lipid moieties were initially prepared based on the Martini (Marrink et al., 2004) topology parameters for cysteine and the membrane lipids POPG and POPE, which are used by, respectively, Lgt and Lnt as substrates to modify the invariant cysteine. Therefore, to create diacylcysteine, the glycerol, *sn*-1 palmitoyl and *sn*-2 oleoyl parameters were taken from POPG and connected to the side-chain particle of cysteine (Figure S2A). To then create the triacylcysteine coordinates the diacylcysteine parameters were modified to connect a palmitoyl tail to the backbone particle of cysteine (Figure S2B). CHARMM-GUI was used to build the atomistic parameters. The tripalmitoyl-modified cysteine parameters in CHARMM-GUI (“CYSL”) were used as the template to build the palmitoyl and oleoyl containing tri- and diacylcysteines (Jo et al., 2017). All parameters are included within CG2AT, to permit conversion of the lipoprotein modifications to an atomic description (Stansfeld and Sansom, 2011) (Figure S2). Finally, virtual sites were developed and added to the cysteine PTMs, as described using the methodology developed for CHARMM36 lipid parameters (Olesen et al., 2018). Parameters for both Martini (versions 2.2 and 3) and CHARMM36 force field cysteine PTMs, with and without virtual sites (Olesen et al., 2018), are available in the Supplemental Information (Data S4).

#### Modelling the Four Stages of Lipoprotein Maturation and Membrane Insertion

The experimentally resolved core domain of the protein was oriented such that the most N-terminal residue was below the core domain, with the Cα atoms of the first and second residues aligned along the z-axis (Figure 2). Therefore the core domain is furthest from the membrane prior to the start of the molecular simulations. In each case the SP was modelled as a helix from its first residue to its lipobox cysteine and again aligned along the z-axis. The distance between the cysteine and the first residue of the core domain was set based on the number of absent residues, with an unstructured length of 3.5 Å per residue. Modeller (Sali and Blundell, 1993) was used to build the missing linker residues, whilst maintaining the distance, orientation and secondary structure of the signal helix. Based on previous NMR structures of BamE (Knowles et al., 2011), Pal (Parsons et al., 2006) and LpoA (Jean et al., 2014) and LpoB (Egan et al., 2014), it is expected that the linkers to the lipoprotein tether will be unstructured and highly dynamic, and therefore it is appropriate to model the missing residues as disordered. Full-length molecular models of lipoproteins were obtained by adding an N-terminal SP helix and tethering loop portion to each template PDB structure through automated comparative modelling by Modeller (Sali and Blundell, 1993).

#### Lipoprotein CG MD Simulations

Lipoprotein membrane assembly simulations were performed using GROMACS and the Martini 2.2 force field (de Jong et al., 2013), with each symmetrical membrane bilayer, comprising POPE and POPG at a 4:1 ratio, assembled based on the pipeline employed for MemProtMD (Stansfeld et al., 2015). The lipoprotein-membrane systems were solvated by 0.15 M NaCl and simulated for 1 μs each for five repeats. For LolB, MlaA and PqiC the molecular systems were run for 25 replicates of 1 μs each. A further set of 25 repeats of 1 μs simulations for LolB were performed with the Martini 3 force field for comparison.

An elastic network model (ENM) was applied to all backbone particles within a cut-off distance of 0.7 nm to model secondary and tertiary structure (Atilgan et al., 2001). The bond lengths were constrained to equilibrium lengths using the LINCS algorithm (Hess et al., 1997). Lennard-Jones and Coulombic interactions are cut off at 1.1 nm, with the potentials shifted to zero at the cut-off (de Jong et al., 2016).

All systems were subjected to steepest-descent energy minimized to remove the initial close contacts, and equilibrated for 1 ns with the protein backbone particles restrained in NPT constant CG-MD simulations. A timestep of 20 fs was used. The neighbour list was updated every 20 steps using the Verlet neighbour search algorithm. The systems were subject to pressure scaling to 1 bar using Parrinello-Rahman barostat (Parrinello and Rahman, 1981), with temperature scaling to 323 K using the velocity-rescaling method (Bussi et al., 2007) with coupling times of 1.0 and 12.0 ps, respectively.

Lipid-protein interactions were calculated based on a 6 Å cut-off for all residues with the proteins. For comparison of protein-lipid interactions, a Pearson's correlation coefficient was calculated based on the similarity between two simulations of a given lipoprotein. Molecular systems were visualised using VMD (Humphrey et al., 1996) and PyMOL (The PyMOL Molecular Graphics System, 2015), with analysis performed using GROMACS tools (Abraham et al., 2015) and MDAnalysis (Michaud-Agrawal et al., 2011).

#### Conversion of Coarse-Grained Systems to Atomistic

Through developments to our CG2AT methodology we are able to convert each CG system to atomistic representation. In addition to the novel methodology for converting the modified cysteine residues, we also implement a hybrid method for the protein structure. This method combines alignment of the structured core of the lipoprotein with the CG coordinates from the final snapshot of the CG simulation. Meanwhile the atomistic details for the N-terminal tether is grown in directly from the CG coordinates, using the Cα-to-main-chain reconstruction method PD2 (Moore et al., 2013) (Figure S2) This enables starting configurations for atomic-level simulations to be constructed from the CG lipoprotein simulations, whilst taking account of the structural changes of the flexible tether to the membrane. Atomistic coordinates for all systems are included in the Supplemental Information for both the monomeric systems and lipoprotein complexes.

#### Modelling the LolB Open Conformation

The open conformation of LolA (PDB ID: 2ZPD) (Oguchi et al., 2008) was used as a template to create an open-state model of LolB, using Modeller (Sali and Blundell, 1993), with its closed structure (PDB ID: 1IWM) (Takeda et al., 2003) used as a guide.

#### Potential of Mean Force Calculations

The PMF calculations were performed using the latest Martini 3 force field. POPE:POPG membranes, at a 4:1 ratio, were configured using *insane* (Wassenaar et al., 2015) to make sure an identical number of lipids were in the both IM leaflets and the inner leaflet of the OM. LPS-REMP molecules were added to the outer leaflet of the OM at 1.7 nm^2^ per LPS (Hsu et al., 2017a). Pulling simulations were performed to create starting points for the umbrella windows. For each system, a force of 1000 kJ mol^-1^ nm^-2^ in the positive z-axis direction was applied to the backbone particle of the cysteine-lipid moiety to pull the molecule away from its equilibrated position in either a membrane or a protein cavity. A second pull was then performed to pull the cysteine deeper into the membrane or protein, so that the centre-of-mass difference between the two was 0. From these steered MD simulations a series of between 50 and 80 frames were extracted, with relative distances between the modified cysteine and the bilayer or protein spaced at consecutive 0.1 nm intervals. This prepares individual windows as the input for the 1 μs umbrella sampling simulations at 310 K. For the LolA and LolB systems, a position-restraining force of 1000 kJ mol^-1^ nm^-2^ in x-, y-, and z-directions was applied to three specific low-mobility backbone beads forming vertices of a triangle at the base of each protein. The restraints ensured optimal location and orientation of the protein without greatly limiting flexibility. Equivalent systems were configured for the diacyl-cysteine moiety. Errors on each free energy profile were estimated using the Bayesian bootstrapping method (Hub et al., 2010). Convergence was assessed by comparing free energy profiles computed from consecutive fractions of simulation time (Figure S7). Three independent sets of simulations were initiated between the cysteine-lipid and LolA, LolB, or the membrane. Each umbrella window was run for 1 μs. PMF free energy landscapes were computed from the three independent sets of windows using the weighted histogram analysis method within GROMACS (Hub et al., 2010).

#### Models and Simulations of the LolCDE Transporter and Its Complex with LolA

Preliminary molecular models of the LolCDE transporter were built using Swiss-model (Waterhouse et al., 2018) and refined using Modeller (Sali and Blundell, 1993) based on structures of MacB in its resting (PDB ID: 5WS4) (Okada et al., 2017) and activated (PDB ID: 5LIL) (Crow et al., 2017) states, and in combination with the soluble complex of LolC and LolA (PDB ID: 6F3Z). LolA was added to the LolCDE complexes, using Modeller, based on the LolC:LolA complex (PDB ID: 6F3Z) (Kaplan et al., 2018). POPG:POPE:Cardiolipin lipid membranes, at a 7:2:1 ratio, were configured using the MemProtMD pipeline (Stansfeld et al., 2015), with CG molecular simulations run for 5 repeats of 5 μs using GROMACS (Abraham et al., 2015).

The end snapshots were converted to an atomistic resolution, with three repeats of 100 ns MD simulation performed using the CHARMM36 force field, for the LolCDE and LolACDE complexes, in both resting and activated conformations; totalling 1.2 μs of atomistic simulation for the Lol transporter (Huang et al., 2017, Klauda et al., 2010).

### Quantification and Statistical Analysis

Membrane interactions were assessed by using a 6 Å cut-off between protein and lipid. Bootstrap analyses were performed to estimate the error bars for the potential of mean force calculations using the Weighted Histogram Analysis Method as implemented in Gromacs. For comparison of protein-lipid interactions, a Pearson’s correlation coefficient was calculated based on the contact profiles between two simulations of a given lipoprotein, implemented and calculated using Python.

### Data and Code Availability

Coordinates of the final models generated by this study are available as a Supplemental Information. All software used in this study is publicly available as stated in the Key Resources Table. Code for setting up and analysing PMF calculations are available from https://github.com/pstansfeld/umbrella_sampling; for adding post-translation modifications to lipoproteins is available from https://github.com/pstansfeld/lipoprotein_ptm; and for converting lipoproteins from CG to atomistic is available from https://github.com/pstansfeld/cg2at.

## Supplemental Information

Document S1. Figures S1–S9

Table S1. Assessment of *E. coli* K12 Lipoprotein Structures, Related to Figure 3 Further details of all 114 predicted lipoprotein sequences, highlighting proteins for which there is an experimentally derived structure available, and those for which it was possible to generate and simulate an accurate molecular model. ^%^Long linker; ^#^Partially modeled; ™Contains transmembrane helices. ^+^Experimental structure available but a more complete model could be generated from a homolog. The PDB IDs for *E. coli* structures are highlighted in bold.

Data S1. Final Coordinates from Simulations (Y–Z), Related to Figure 3

Data S2. Final Coordinates from Simulations (A–L), Related to Figure 3

Data S3. Final Coordinates from Simulations (M–X), Related to Figure 3

Data S4. Parameter Files for Lipid Modifications, Related to Figure 2

Document S2. Article plus Supplemental Information
