# Supplementary material for: Characterizing Membrane Association and Periplasmic Transfer of Bacterial Lipoproteins through Molecular Dynamics Simulations
Source: Structure. 2020 Apr 7;28(4):475–487.e3. doi: 10.1016/j.str.2020.01.012 (PMC7139219; doi:10.1016/j.str.2020.01.012)
Supplement: Document S1. Figures S1–S9 [file mmc1.pdf]

Structure, Volume *28*

Supplemental Information

Characterizing Membrane Association and Periplasmic Transfer of Bacterial Lipoproteins through Molecular Dynamics Simulations

Shanlin Rao, George T. Bates, Callum R. Matthews, Thomas D. Newport, Owen N. Vickery, and Phillip J. Stansfeld

Structure

Supplementary Information

Characterising Membrane Association and Periplasmic Transfer of Bacterial Lipoproteins through Molecular Dynamics Simulations

Shanlin Rao, George T. Bates, Callum R. Matthews, Thomas D. Newport, Owen N. Vickery, and Phillip J. Stansfeld

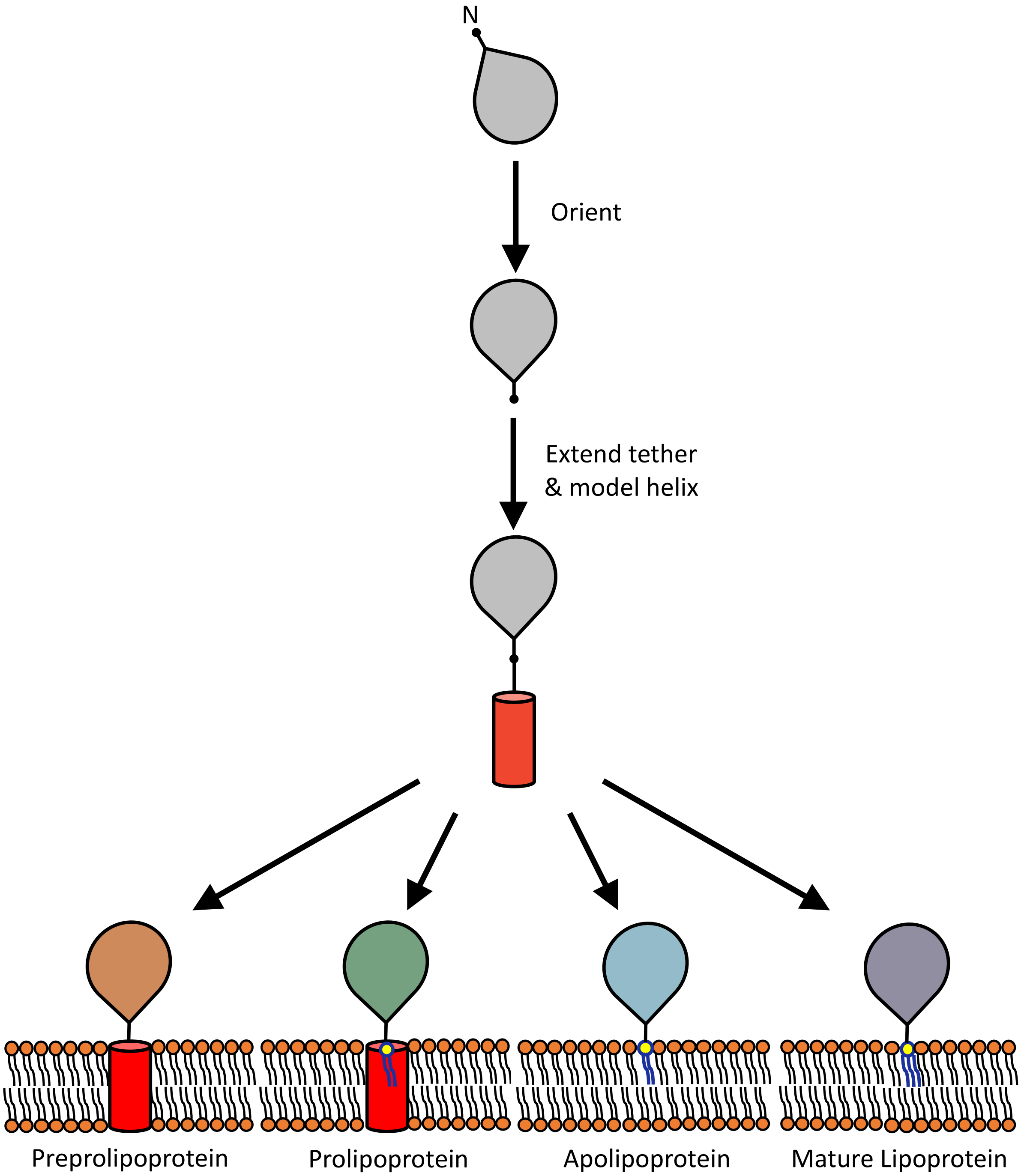

Figure S1: Schematic of the Modelling Process for Lipoproteins. Related to Figure 2. Lipoprotein structures are oriented so that the N-terminal end of the protein are directed towards the membrane. A helix and missing atoms are modelled linearly below the N-terminus. The lipoprotein is integrated into a membrane as either a preprolipoprotein, prolipoprotein, apolipoprotein or mature lipoprotein.

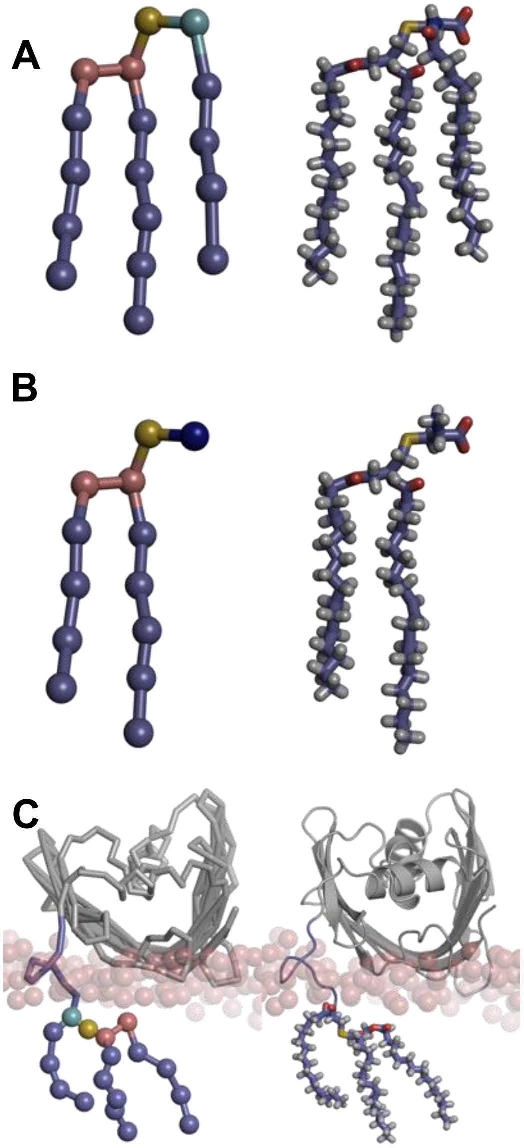

Figure S2: Molecular Parameters for CG and AT Lipoprotein PTM Anchors. Related to Figure 2.

Martini v2 CG and CHARMM36 AT coordinates for (A) triacylated cysteine and (B) diacylated cysteine. (C) Example conversion from CG to AT coordinates for LolB as a triacylated lipoprotein. The triacyl-cysteines and missing tethers from the X-ray structure are coloured blue, with the remainder of the structures coloured grey. Phosphate particles and atoms are shown as spheres and coloured red. Here the oleyl group is shown with five beads for the CG model of the post-translational modifications. Extended data also contains coordinates and parameters for the 4-bead modification.

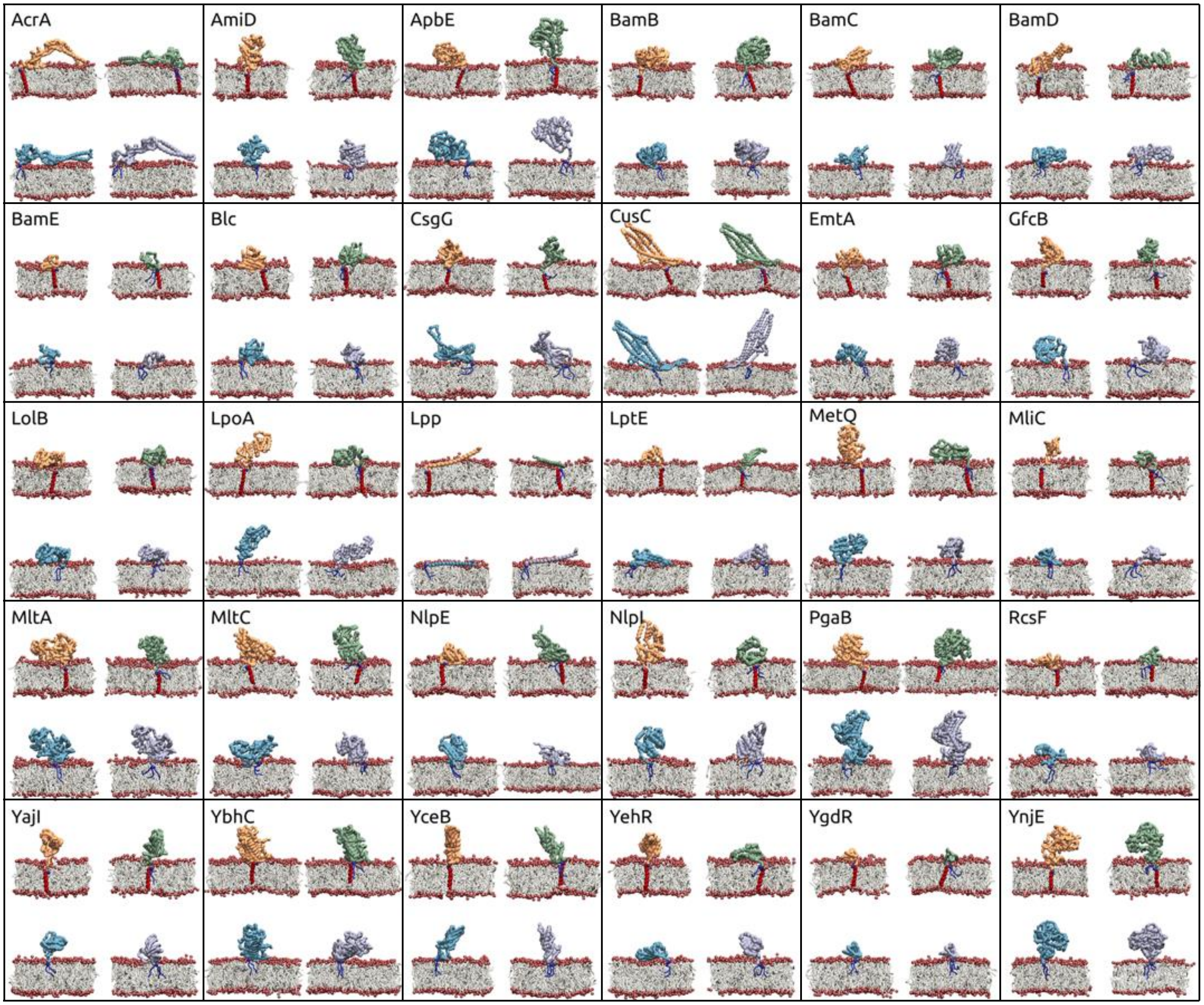

Figure S3: Simulation Snapshots from Monomeric Lipoprotein Maturation Stages. Related to Figure 2.

Coarse-grained molecular simulations of 30 molecular structures of the 114 predicted *E. coli* lipoproteins. The membrane association with the four discrete membrane anchors is shown.

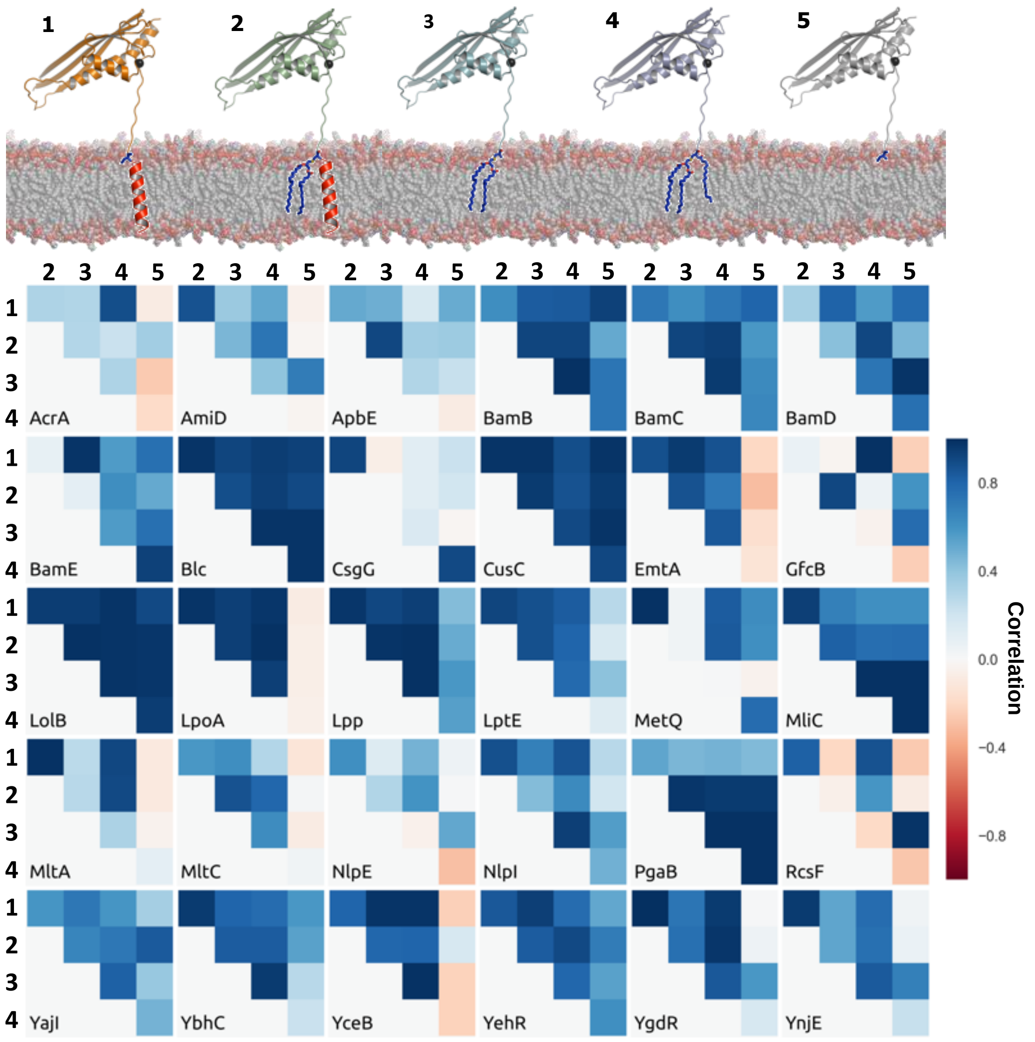

Figure S4: Comparison of Membrane-Protein Interactions for all 30 Structures. Related to Figure 2.

For all 30 structures the interactions between the membrane and the lipoproteins are compared for all four maturation states, from left to right, SP (1), SP with diacyl-cysteine (2), diacyl-cysteine (3) and triacyl-cysteine (4). A fifth configuration is simulated, representing a non-physical state where there is no membrane anchor (5). In each case the protein-membrane interactions are compared, with a Pearson correlation coefficient calculated to assess the similarity in residue-lipid interactions. The correlation coefficient value is coloured on a red-white-blue scale from -1 to 1, where a value of 1 has identical residue-lipid interactions, and a value of -1 suggests a distinct set of contacts.

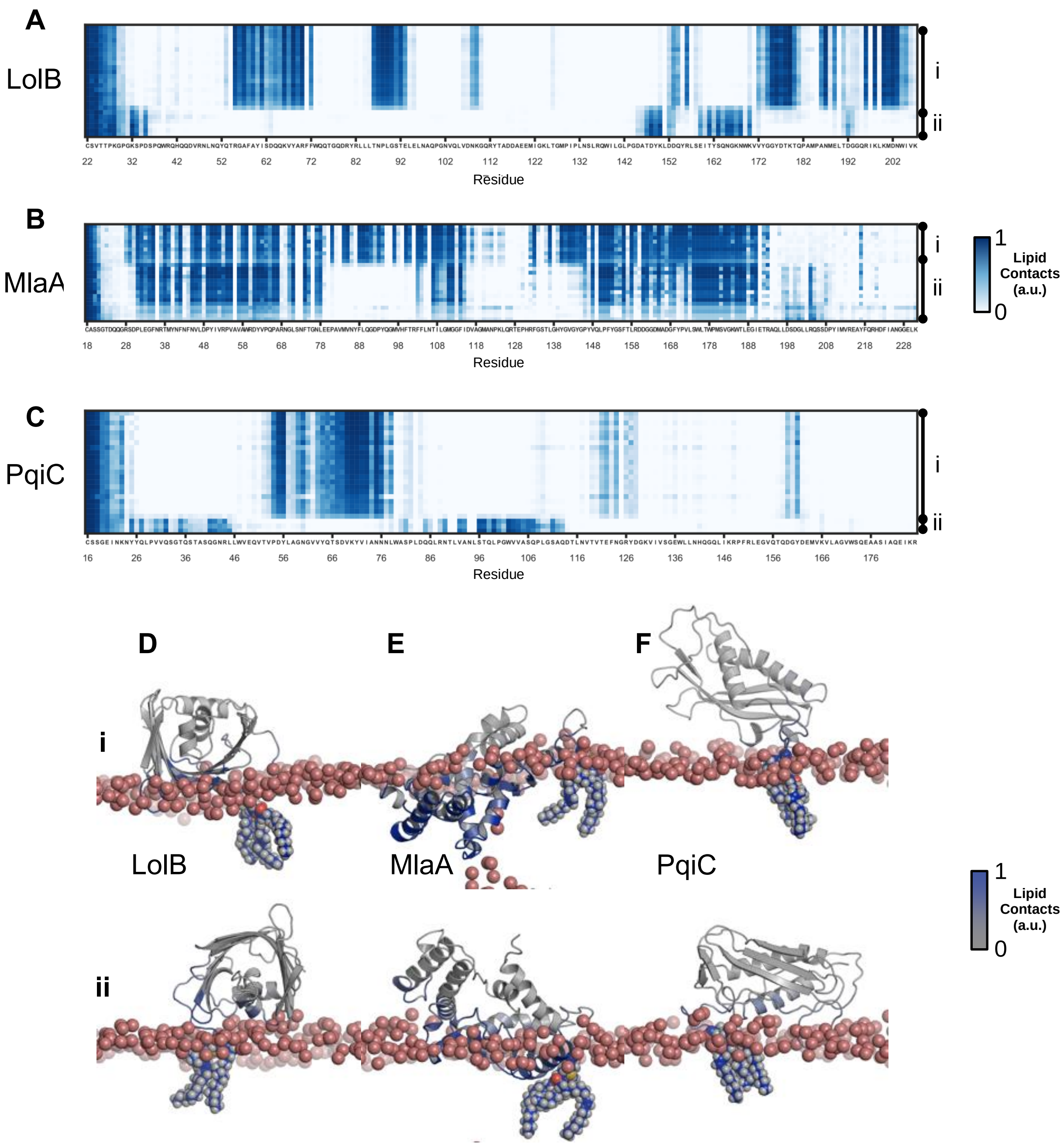

Figure S5: Lipid Contacts of LolB, MlaA and PqiC. Related to Figure 6.

Membrane contacts of **(A)** LolB, **(B)** MlaA and **(C)** PqiC over 25 repeats of the 1 μs CG molecular simulation and coloured on a white to blue scale, with blue indicating extensive lipid contacts. The lipid-residue contacts for the two binding modes, **(i)** and **(ii)**, shown in Figure 6 are annotated for each replicate. Molecular images of the membrane association of **(D)** LolB, **(E)** MlaA and **(F)** PqiC in their **(i)** primary and **(ii)** secondary binding orientations after 100 ns of atomistic molecular simulation. Phosphate atoms are shown as red spheres. Proteins are shown as a cartoon representation, and coloured on a white to blue scale.

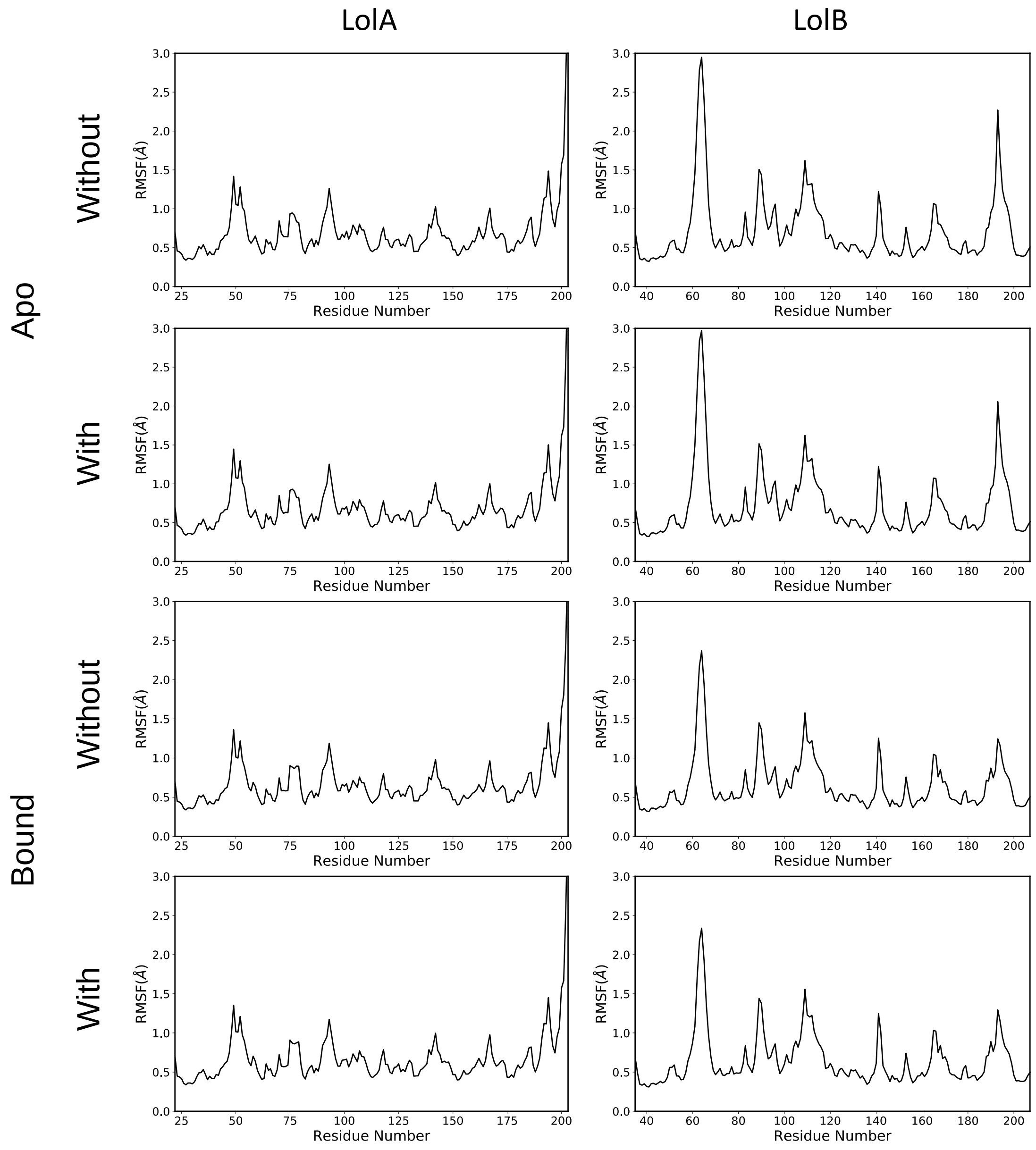

Figure S6. The triangular position restraints do not influence LolA and LolB dynamics. Related to Figure 7.

The coarse-grained dynamics of LolA and LolB with and without bound triacyl cysteine, and with and without the triangulated positional restraints. The restraints have limited impact on the root mean square fluctuations, while the presence of the triacyl cysteine stabilises regions at the N- and C- termini of LolB, but has limited influence on the dynamics of LolA.

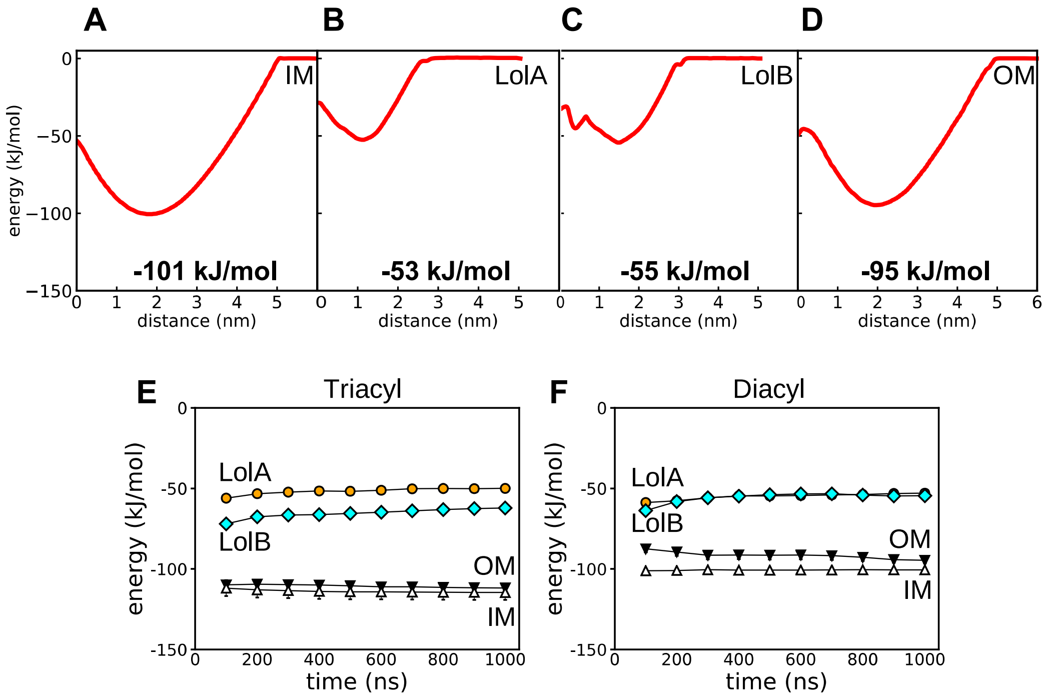

Figure S7: Diacyl-cysteine PMFs and Convergence Analysis. Related to Figure 7. The energetics associated with diacyl-lipoprotein transfer across the periplasm, obtained from umbrella sampling and PMF calculations, calculated using WHAM with errors computed using Bayesian bootstrapping. Plots are shown for the extraction of the diacyl-cysteine moiety from the **(A)** IM, **(B)** LolA, **(C)** LolB, and **(D)** OM. The annotated energy values are derived from the minimum value of the PMF. Minimum free energy values calculated by taking incremental 100 ns subsections from the PMF calculations for **(E)** triacyl-cysteine and **(F)** diacyl-cysteine. The plots indicate that by 1 μs the free energy values have suitably converged.

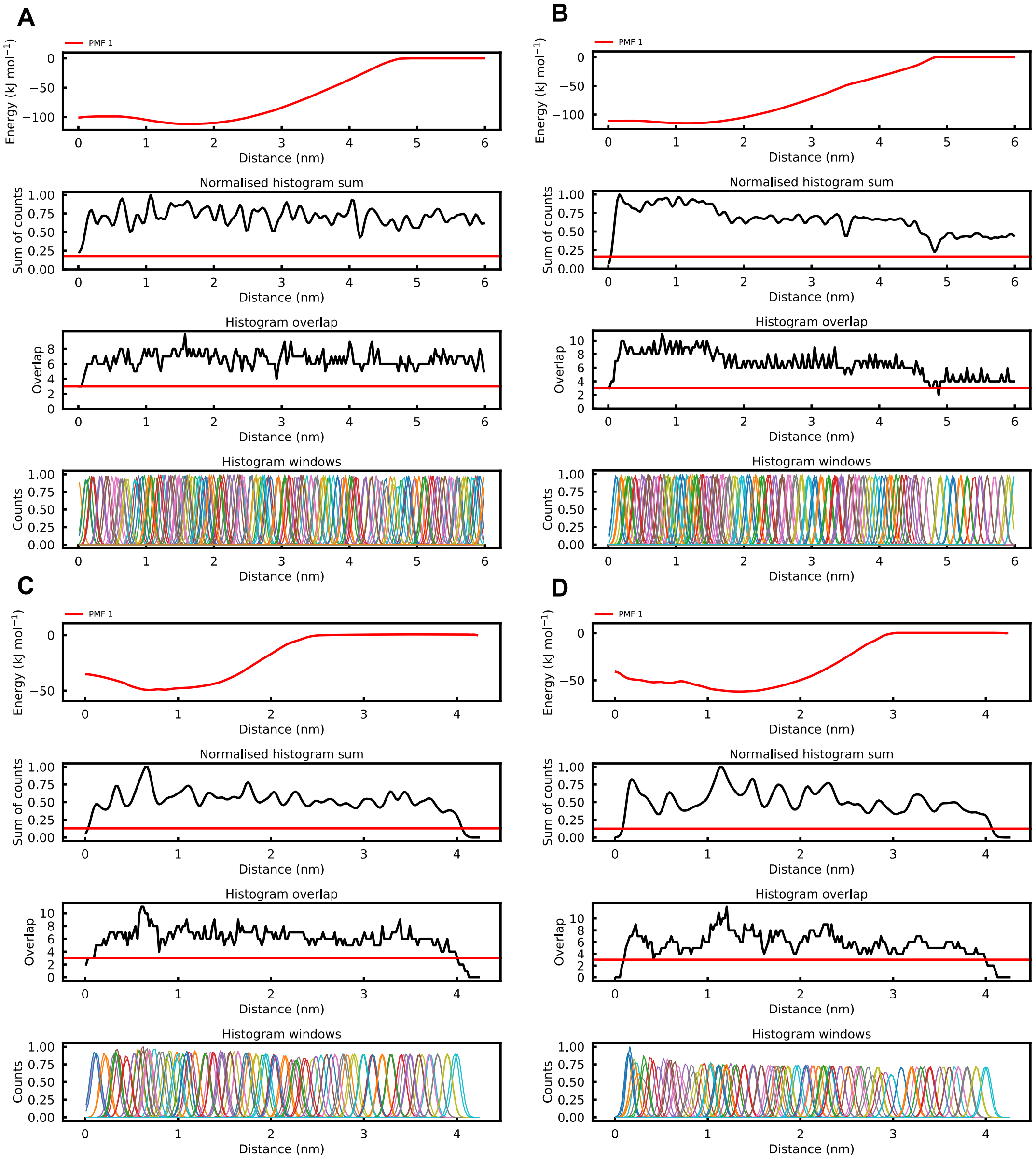

Figure S8. Histogram data for the triacyl-cysteine PMFs. Related to Figure 7. Properties of the PMF calculations for the binding of the triacyl-cysteine to **(A)** IM, **(B)** OM, **(C)** LolA and **(D)** LolB. For each dataset the PMF curve is shown alongside details of the histogram counts and overlap for each distance between bound and unbound.

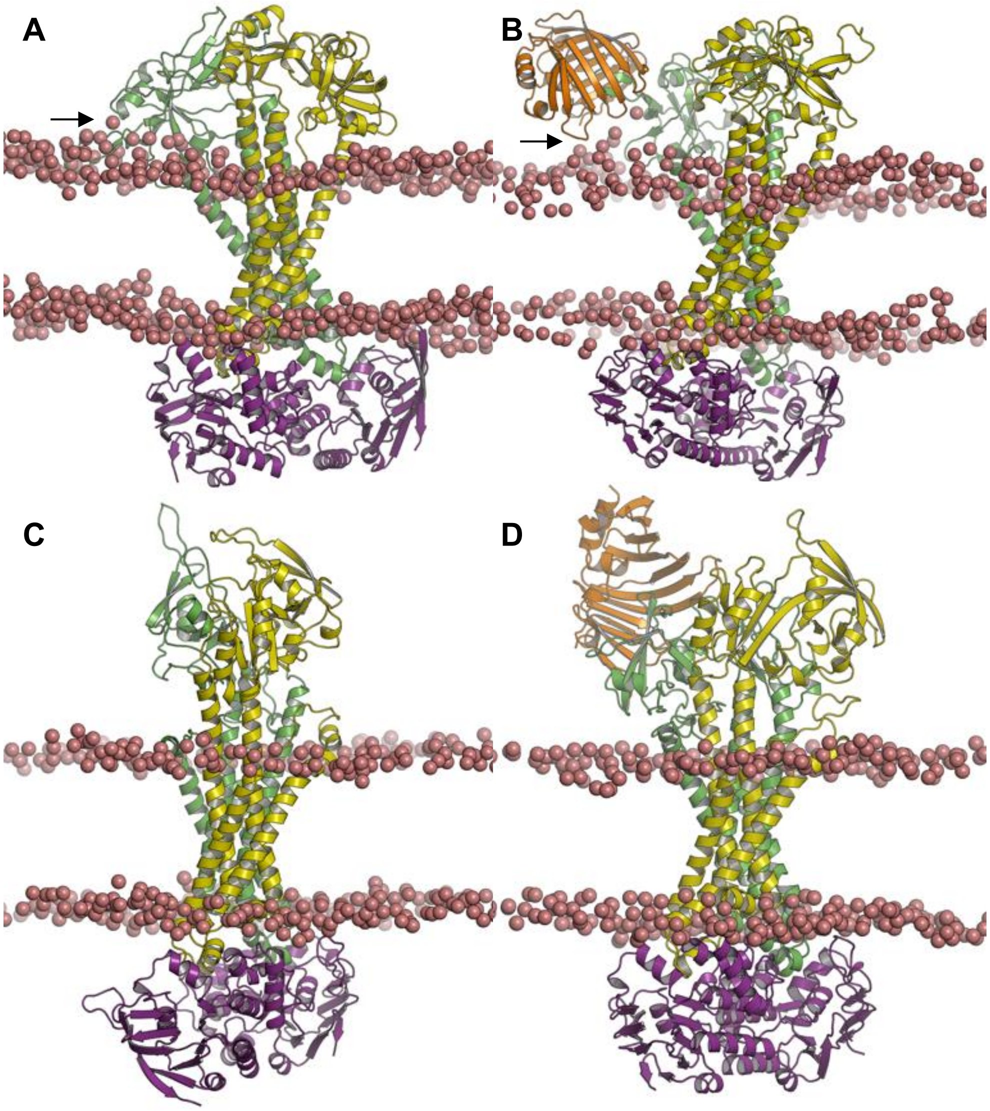

Figure S9. Atomistic molecular simulations of the Lol ABC transporter. Related to Figure 8.

In the resting states of the **(A)** LolCDE, and **(B)** LolACDE transporters, the membrane deformation from the CG simulations is retained about the periplasmic domain of LolC (green; black arrow), with this domain tightly associated with the membrane. This association also brings LolA (orange) in close proximity to the membrane for lipoprotein transfer. The planar membranes that were observed in the CG simulations of the activated forms of **(C)** LolCDE, and **(D)** LolACDE also show no deformation at the atomistic level of simulation. Phosphorus atoms of the membrane are shown as red spheres. LolD is coloured purple, while LolE is shown in yellow.
